# Beyond Janzen's Hypothesis: How Amphibians That Climb Tropical Mountains Respond to Climate Variation

**DOI:** 10.1093/iob/obad009

**Published:** 2023-05-03

**Authors:** R P Bovo, M N Simon, D B Provete, M Lyra, C A Navas, D V Andrade

**Affiliations:** Departamento de Fisiologia, IB, Universidade de São Paulo, São Paulo, SP 05508-090, Brasil; Departament of Integrative Biology, Oklahoma State University, Stillwater, OK 74018, USA; Departament of Integrative Biology, Oklahoma State University, Stillwater, OK 74018, USA; Instituto de Biociências, Universidade Federal de Mato Grosso do Sul, Campo Grande, MS 79070-900, Brasil; Departamento de Biodiversidade, IB, Universidade Estadual Paulista, Rio Claro, SP 13506-900, Brasil; New York University Abu Dhabi, Saadiyat Island, Abu Dhabi 129188, United Arab Emirates; Departamento de Fisiologia, IB, Universidade de São Paulo, São Paulo, SP 05508-090, Brasil; Departamento de Biodiversidade, IB, Universidade Estadual Paulista, Rio Claro, SP 13506-900, Brasil

## Abstract

Janzen's hypothesis (JH) posits that low thermal variation selects for narrow physiological tolerances, and thus small species distributional ranges and high species turnover along tropical elevational gradients. Although this hypothesis has been intensely revisited, it does not explain how many tropical species may exhibit broad distributions, encompassing altitudinal gradients. Moreover, the physiological responses of tropical species remain largely unknown, limiting our understanding on how they respond to climate variation. To fill these knowledge gaps, we tested a major component of JH, the climate variability hypothesis (CVH), which predicts broader thermal tolerance breadth (Tbr = CTmax – CTmin) with broader temperature variation. Specifically, we sampled populations of five amphibian species distributed in two mountain ranges in Brazil's Atlantic Forest to test how CTmin and CTmax vary along elevational gradients. Since both thermal and water balance traits are pivotal to the evolutionary history of amphibians, we also measured rates of dehydration and rehydration and their relations with thermal tolerances. We found that broader temperature variation with increasing altitude did not always lead to broader Tbr, since changes in CTmin and CTmax were species-specific. In addition, we found that water balance did not show consistent variation with altitude, also with low correlations between hydric and thermal traits. While we also found that highland populations are at lower risk of thermal stress than lowland counterparts, both are living far from their upper thermal limits. As a consequence of intraspecific variation in physiological traits and spatial variation in climate along altitude, responses to climate variation in tropical amphibian species were context-dependent and heterogeneous. Together with recent studies showing thermal tolerances of some tropical amphibians comparable to temperate taxa, our findings highlight that several responses to climate variation in tropical species may not conform to predictions made by either the CVH or other important hypotheses concerning physiological variation. This reinforces the need to overcome geographical bias in physiological data to improve predictions of climate change impacts on biodiversity.

**(Portuguese abstract) Resumo** A Hipótese de Janzen (JH) postula que a baixa variação térmica seleciona tolerâncias fisiológicas estreitas e, portanto, amplitudes restritas de distribuição das espécies e alta substituição de espécies ao longo de gradientes altitudinais tropicais. Embora intensamente revisitada, essa hipótese não explica como espécies tropicais podem exibir amplas distribuições geográficas, abrangendo gradientes altitudinais. Além disso, as respostas fisiológicas das espécies tropicais permanecem amplamente desconhecidas, limitando nossa compreensão sobre como elas respondem à variação climática. Para preencher essas lacunas de conhecimento, testamos um componente importante da JH, a Hipótese de Variabilidade Climática (CVH), que prevê uma maior amplitude de tolerância térmica (Tbr = CTmax – CTmin) quando a variação da temperatura ambiental é mais ampla. Especificamente, amostramos populações de cinco espécies de anfíbios distribuídas em duas cadeias montanhosas na Mata Atlântica do Brasil para testar como CTmin e CTmax variam ao longo de gradientes de altitude. Dado que parâmetros térmicos e do balanço hídrico são fundamentais para a história evolutiva dos anfíbios, também medimos as taxas de desidratação e reidratação e suas relações com as tolerâncias térmicas. Encontramos que uma variação de temperatura ambiental mais ampla com o aumento da altitude nem sempre conduz a uma Tbr mais ampla, uma vez que as mudanças em CTmin e CTmax foram espécie-específicas. Além disso, encontramos que o balanço hídrico não apresentou variação consistente com a mudança de altitude, e que as correlações entre parâmetros hídricos e térmicos foram baixas. Embora populações das maiores altitudes apresentaram menor risco de estresse térmico do que populações da mesma espécie em altitudes menores, ambas estão vivendo longe de seus limites térmicos superiores. Em consequência da variação intraespecífica em parâmetros fisiológicos e variação espacial no clima ao longo da altitude, as respostas à variação climática em espécies de anfíbios tropicais foram contexto-dependentes e heterogêneas. Juntamente com estudos recentes indicando tolerâncias térmicas de alguns anfíbios tropicais comparáveis a de táxons temperados, nossas descobertas destacam que várias respostas à variação climática em espécies tropicais podem não estar de acordo com as previsões feitas pela CVH ou outras hipóteses importantes sobre a variação fisiológica. Isso reforça a necessidade de superar o viés geográfico em dados fisiológicos para aperfeiçoar previsões dos impactos das mudanças climáticas sobre a biodiversidade.

**(Spanish abstract) Resumen** La hipótesis de Janzen (JH) postula que la baja variación térmica selecciona tolerancias fisiológicas estrechas y, por lo tanto, rangos de distribución de especies restringidos con alta rotación de especies a lo largo de gradientes de elevación tropicales. Aunque esta hipótesis ha sido intensamente discutida, no explica cómo várias especies tropicales pueden exhibir distribuciones amplias, abarcando gradientes altitudinales. Además, las respuestas fisiológicas de las especies tropicales siguen siendo bastante desconocidas, lo que limita la comprensión de cómo responden a la variación climática. Para llenar estos vacíos de conocimiento, examinamos un componente importante de JH, la Hipótesis de Variabilidad Climática (CVH), que predice mayor amplitud de tolerancia térmica (Tbr = CTmax – CTmin) cuando la variación de temperatura es más amplia. Específicamente, tomamos muestras de poblaciones de cinco especies de anfibios distribuidas en dos cadenas montañosas en el Bosque Atlántico de Brasil para verificar cómo CTmin y CTmax varían a lo largo de este gradiente de elevación. Dado que los rasgos de equilibrio térmico y hídrico son fundamentales para la historia evolutiva de los anfibios, también medimos las tasas de deshidratación y rehidratación y sus relaciones con las tolerancias térmicas. Encontramos que una variación de temperatura más amplia con el aumento de la altitud no siempre conduce a una Tbr más amplia, ya que los cambios en CTmin y CTmax son específicos de la especie. Además, encontramos que el balance hídrico no muestra variación consistente con la altitud, con bajas correlaciones también entre los rasgos hídricos y térmicos. Si bien las poblaciones de las tierras altas tienen un menor riesgo de estrés térmico que las contrapartes de las tierras bajas, ambas se encuentran lejos de sus límites térmicos superiores. Como consecuencia de la variación intraespecífica en los rasgos fisiológicos y la variación espacial en el clima a lo largo de la altitud, las respuestas a la variación climática en las especies de anfibios tropicales fueron dependientes del contexto y heterogéneas. Junto con estudios recientes que muestran tolerancias térmicas de algunos anfibios tropicales comparables a los taxones de zonas templadas, nuestros hallazgos resaltan que varias respuestas a la variación climática en especies tropicales pueden no ajustarse a las predicciones hechas por el CVH u otras hipótesis importantes sobre la variación fisiológica. Esto refuerza la necesidad de superar el sesgo geográfico en los datos fisiológicos para mejorar las predicciones de los impactos del cambio climático en la biodiversidad.

## Graphical Abstract

**Graphical Abstract:**
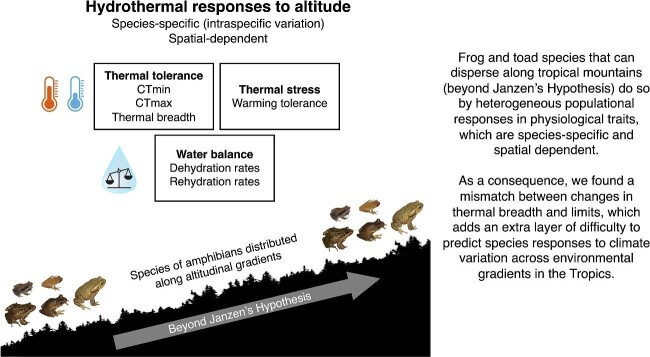

## Introduction

Janzen's hypothesis (JH) postulates the physiological tolerances of tropical species to be narrower than of temperate species, reflecting lower seasonal overlap of thermal regimes along elevational gradients. In turn, this would lead to more effective dispersal barriers, limiting gene flow across altitude, and thus high species turnover in tropical mountains (Janzen 1967). Although JH has been intensely revisited over the years (Ghalambor et al. 2006; Shah et al. 2017; Polato et al. 2018; Sheldon et al. 2018; Mammola et al. 2019), it does not explain how some tropical species can exhibit broad distributions, encompassing environmental gradients, such as lowlands and highlands. To better understand how can such species respond to the climate variation imposed by elevational gradients, we tested hypotheses of how physiological traits would vary in tropical species distributed along altitude.

We tested one of the components of JH, the climate variability hypothesis (CVH; Dobzhansky 1950; Pither 2003; Bozinovic et al. 2011). The CVH predicts that wider thermal variation results in a broader thermal tolerance breadth, the difference between critical thermal maximum and minimum (Tbr = CTmax – CTmin) (Table 1). There are two hypotheses that can be used to predict how thermal breadth might shift across temperature gradients (e.g., latitude, altitude), the heat-invariant and the cold-variability hypotheses (Table 1). The heat-invariant hypothesis (Brett 1956; Bozinovic et al. 2014; Pintanel et al. 2019) states that CTmax will show little (if any) change with the thermal environment, whereas the cold-variability hypothesis (Araújo et al. 2013; Sunday et al. 2014) predicts CTmin to show larger changes with thermal variation. This difference in degree of response between CTmax and CTmin may be due to non-excluding explanations, including higher physiological and evolutionary constraints on CTmax compared with CTmin (Araújo et al. 2013; Hoffmann et al. 2013; Sunday et al. 2014), behavioral buffering (Bogert 1949; Huey et al. 2003; Muñoz 2022), and timing of activity (Wells 2007; Araújo et al. 2013) that may expose organisms to differential selection on thermal tolerance limits. The predicted lower change of CTmax compared to CTmin along climatic gradients (e.g., latitude or altitude) could also impact organismal responses to thermal stress, when organisms are exposed to wide temperature variation negatively affecting their performance (Deutsch et al. 2008). Thus, if CTmax is similar between lowland and highland counterparts, but maximum environmental temperature (Tmax) is higher at lower elevations, then lowland inhabitants would be living closer to their CTmax and show higher thermal stress (Tewksbury et al. 2008; Huey et al. 2009; Sunday et al. 2014).

**Table 1:** Summary of hypotheses tested in our study.

Hypotheses	Definition	Study predictions	Study results
Climate variability hypothesis (CVH)	Broader environmental temperature variation will lead to broader thermal tolerance breadth, the difference between critical thermal maximum and minimum (Tbr = CTmax – CTmin).	Increased temperature variation with altitude would select for broader TBr within-species.	Partially agree
Heat-invariant hypothesis	CTmax will show little (if any) changes with increases in maximum environmental temperatures.	CTmax will show little (if any) decrease in populations at highlands than at lowlands.	Generally agree
Cold-variability hypothesis	CTmin will show large changes with decreases in minimum environmental temperatures.	CTmin will be lower and more variable in populations at highlands than at lowlands.	Agree

We used Janzen's hypothesis (JH) as a background to leverage our main question: how can tropical species overcome the predicted physiological barrier to dispersal across altitudes? The climate variability hypothesis (CVH) is a major component of JH. The other two hypotheses can be used to predict how thermal breadth might shift across environmental (temperature) gradients (e.g., latitude, altitude). The magnitude of changes (if little or large) in CTmin and Ctmax are in comparison with each other.

We chose to work with tropical amphibians to test the aforementioned hypotheses for two reasons. First, tropical amphibians comprise many broadly distributed species in tropical mountains, allowing us to sample populations from the same species along the elevational gradient. It is important to notice that these wide distributed tropical species across altitude likely overcame dispersal barriers related to physiological tolerances that would result in limited or no gene flow across altitudes. Second, most amphibians have highly permeable skin and face challenging constraints in terms of water balance regulation (Tracy 1976; Hillman et al. 2009). In fact, thermal and water balance traits are pivotal in the evolutionary history of amphibians, rendering water balance and thermal tolerance indissoluble. Indeed, patterns of physiological variation among different populations can be linked to differences in both thermal and hydric environments (Bozinovic et al. 2011; Buckley and Kingsolver 2021). Therefore, studies addressing physiological responses in amphibians are more robust if they assess thermal and hydric trait variation associated with environmental gradients. Accordingly, we also tested whether rates of evaporative water loss (EWL) and water uptake (WU)—proxies of, respectively, dehydration and rehydration—would change with altitude, going beyond the thermal tolerance traits.

Because highlands tend to be colder, more thermally variable, and drier than lowlands (Körner 2007; Malhi et al. 2010; Polato et al. 2018), we expected highland populations to show (1) lower CTmin and CTmax, but CTmin changing more than CTmax (as predicted by cold-variability and heat-invariant hypotheses), (2) broader thermal breadth (Tbr = CTmax – CTmin) (as predicted by CVH), and (3) lower rates of EWL, but higher rates of WU than their lowland counterparts (as expected by natural responses to drier environments at highlands). In addition, to measure differences in thermal stress between lowland and highland counterparts, we estimated warming tolerance (WT = CTmax – Tmax of the environment, *sensu*Duarte et al. 2012). We expected higher WT, meaning that CTmax is far from Tmax, thus lower thermal stress in highland populations if CTmax decreases less than the decrease in Tmax (e.g., Catenazzi et al. 2014; von May et al. 2017).

We tested the aforementioned predictions in five frog species distributed in mountains in the Brazil's Atlantic Rainforest. Tropical amphibians in South America are a suitable model to study organismal responses to climate variation because one of its most iconic habitats, the Atlantic Rainforest, harbors the highest richness of amphibians worldwide, including a variety of ecologies and physiologies (Haddad et al. 2013). Also, several studies using published datasets of physiological tolerances of amphibians to assess potential responses to climate changes in tropical regions (Sunday et al. 2011, 2014, 2019; Gunderson and Stillman 2015; Pinsky et al. 2019) are geographically biased and largely restricted to species from Central America and Australia (largely based on Brattstrom pioneer studies; Brattstrom 1968, 1970), which could mislead generalizations to large-scale tropics. Hence, working with understudied tropical frogs from Brazil helps to alleviate this geographical bias, filling an important knowledge gap on the potential diversity in the physiological capacity of tropical species to cope with natural climate variation along environmental gradients.

## Material and methods

### Study animals and laboratory maintenance after field collections

**Fig. 1:**
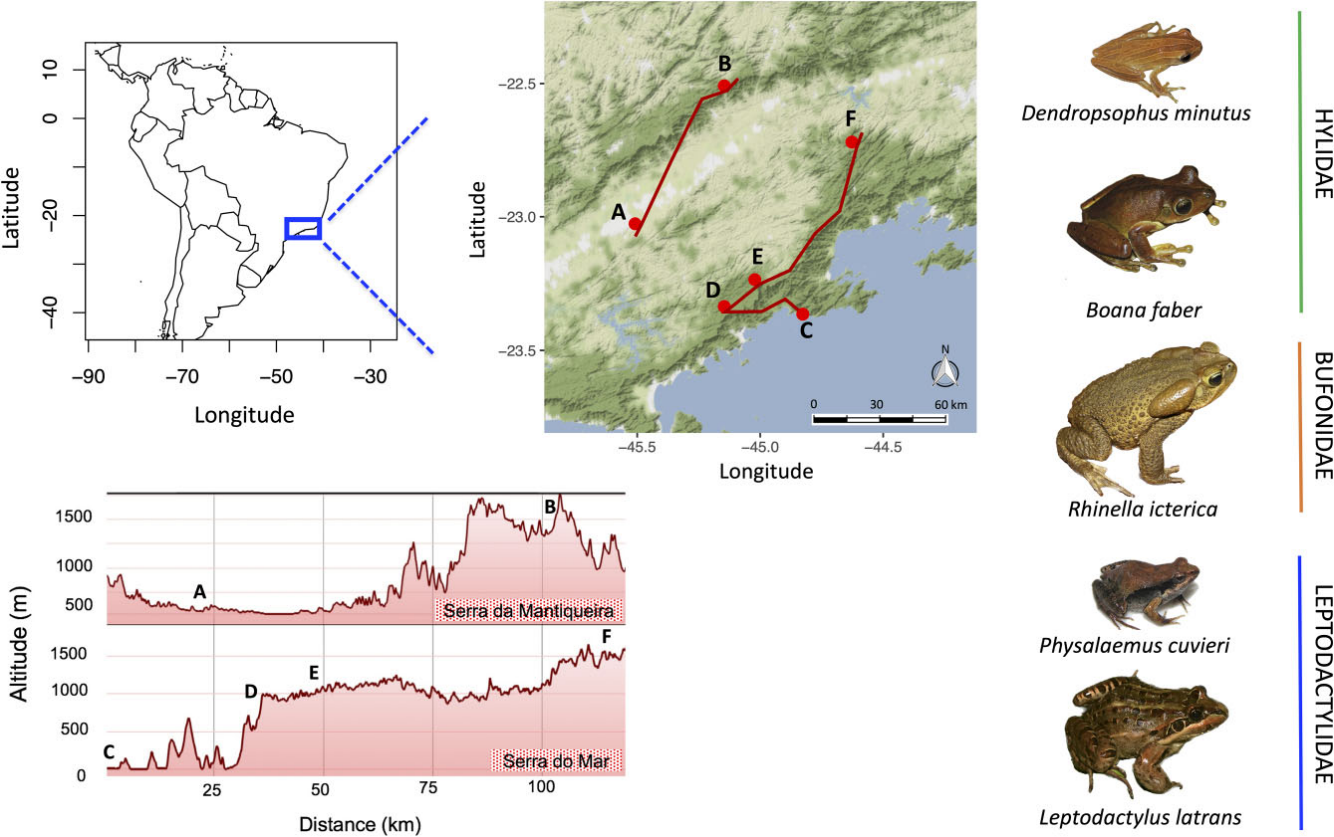
Map (top left) showing the distribution of sampling sites (top right, A–F) in eastern Sao Paulo state in Brazil's Atlantic Forest. *Serra da Mantiqueira* (A: Taubaté 550 m a.s.l., B: Pico dos Marins 1600 m) and *Serra do Mar* (C: Picinguaba 35 m, D: Santa Virginia 820 m, E: Cunha 1022 m, F: Serra da Bocaina 1500 m), where three to five populations of five amphibian species were collected. Altitudinal profile of the two mountain ranges (bottom panel), confirming that our sampling sites encompassed low and high elevations in the Atlantic Forest. Frog pictures are licensed under CC BY-NC, except *Boana faber*, which was photographed by Andrés Brunetti.

We collected 225 adults of five species of amphibians (Table S1) along two tropical elevational gradients—from sea level to 1600 m a.s.l.—in Brazil's Atlantic Forest (Fig. 1) during warm/wet seasons (September to February 2011–2014). We collected individuals from three to five geographical populations (*sensu*Hedrick 2011) of each species (see Section ‘Study site and environmental variation descriptors’; Table S1). All species breed in aquatic habitats but occupy either arboreal (n = 59 for *Dendropsophus minutus*, n = 46 for *Boana faber*; Hylidae), terrestrial (n = 50 for *Rhinella icterica*; Bufonidae), or water margins habitats (n = 31 for *Leptodactylus latrans*, n = 39 for *Physalaemus cuvieri*; Leptodactylidae) (Haddad et al. 2013). We aimed to sample species from different taxonomic affiliations, ecologies (microhabitat use, arboreal to terrestrial to freshwater), morphologies (small and large body sizes, Table S1), and physiologies (main goal of this study). When we conducted fieldwork, the nominal species *L. latrans* comprised all the individuals we sampled, however, a lineage at one location was very recently revalidated as *L. luctator* (Magalhães et al. 2020). Since these two species belong to the same species group with a complex taxonomic history, we treat them as functionally and ecologically equivalent: both inhabit identical microhabitats, have identical life history attributes, and are morphologically similar.

Our field expeditions did not exceed 3 days. Animals were located by acoustic and visual searching at night and then transported to the laboratory, which was 217 to 305 km away from sampling sites. In the laboratory (613 m: –22.397331 S, 47.547799 W), all animals were kept under natural thermal/humidity regimes (daily temperature range of 22–27°C, air humidity of 45–65%) and photoperiod (light/dark cycles 13.5:10.5). They were kept individually in separated plastic terraria (40 × 29 × 13.5 cm) with a polyvinyl chloride (PVC) tube for shelter at one end of the container, and a bowl full of water on the opposite end. To avoid any potential metabolic interference on water balance and thermal tolerances (e.g., Witters and Sievert 2001), we did not feed animals in the laboratory.

### Phylogeographic structure among populations

In the last few years, numerous studies have revealed the existence of cryptic species among tropical amphibians (Fouquet et al. 2007; Vieites et al. 2009; Funk et al. 2012). To ensure that the species were broad distributed along the elevational gradients, and not potential cryptic species, we performed a simplified genetic analysis using the mitochondrial 16S rRNA gene to quantify the phylogeographic structure within species across altitude (Table S2). Details on DNA extraction, RNA amplification, and sequence alignment are in the Supplementary Material 2.

If a phylogeographic structure exists (i.e., highland and lowland populations shown as distinct and deep divergent genetic lineages), it indicates low or no gene flow among them (Slatkin 1987; Medina et al. 2021). Limited gene flow across altitudes, as predicted by JH, would support the concept of dispersal barriers (i.e., the mountain passes higher in the tropics) due to differential physiological tolerances. This scenario would be indicated by the detection of potential cryptic species dispersed along altitude but currently classified as a single species. Conversely, if no genetic-geographic structure is found, or if the structure is not correlated with altitude, gene flow was likely present (at least at some point in the past), confirming that we sampled different populations of the same broad distributed species across altitudes.

We found support for the latter scenario. Lowland and highland populations are not separate clades in our genetic tree in four out of five species (Fig. S1). The only exception was the species *L. latrans* in which a lineage at one location was very recently revalidated as *L. luctator* (see details in Section ‘Study animals and laboratory maintenance after field collections’). Nevertheless, this did not affect our analyses on potential physiological responses to climatic variation with altitude because *L. luctator* is restricted to one of the two mountain ranges we studied (Serra da Mantiqueira; see Section ‘Study site and environmental variation descriptors’), in which we could only sample a population at high elevation (1600 m a.s.l.). In the other mountain range (Serra do Mar; see Section ‘Study site and environmental variation descriptors’), we could sample and compare a lowland (35 m) and a highland (1022 m a.s.l.) population of *L. latrans*. In summary, we studied tropical species that are indeed broadly distributed across altitudes.

### Study site and environmental variation descriptors

We collected animals in four localities at *Serra do Mar*: (i) Serra do Mar State Park, Picinguaba (35 m a.s.l., –23.364525 S, 44.826944 W), (ii) Serra do Mar State Park, Santa Virginia (820 m, –23.336200 S, 45.145917 W), (iii) Serra do Mar State Park, Cunha (1022 m, –23.235556 S, 45.021944 W), (iv) Serra da Bocaina National Park (1500 m, –22.720017 S, 44.627133 W); and two localities at *Serra da Mantiqueira*: (i) Taubaté (550 m, –23.026341 S, 45.508664 W), (ii) Pico dos Marins (1600 m, –22.508533 S, 45.14916  W) (Fig. 1). Both mountain ranges are covered with mesic tropical and subtropical humid forests (Oliveira-Filho and Fontes 2000).

**Fig. 2:**
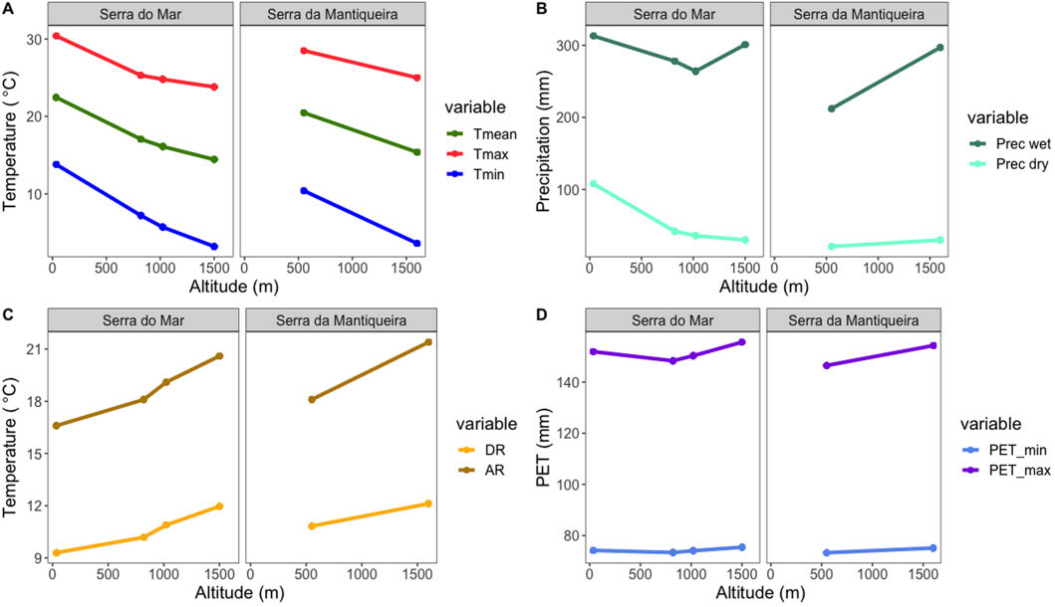
Changes in temperature, rainfall, and potential evapotranspiration (PET; a proxy of aridity, which increases with PET values) along the altitudinal gradients in the two mountain ranges: *Serra do Mar* and *Serra da Mantiqueira*. Minimum temperature of the coldest month (Tmin = BIO_6), mean annual temperature (Tmean = BIO_1), and maximum temperature of the warmest month (Tmax = BIO_5) decrease with altitude (A) in both mountain ranges (despite differences in mean and magnitude). Diurnal (DR = BIO_2) and annual (AR = BIO_7) thermal ranges increase with altitude (C), ranging, respectively, from 9.3°C to 12.1°C, and from 16.6°C to 21.4°C. Precipitation of the wettest (Prec wet = BIO_13) and of the driest month (Prec dry = BIO_14), and potential evapotranspiration (PET minimum of the driest month, PET maximum of the wettest month) tend to remain relatively stable across altitudes in the study locations.

To obtain environmental thermal profiles, we extracted bioclimatic variables from Worldclim 2.1 (Fick and Hijmans 2017): mean annual temperature (Tmean = BIO1), minimum temperature of the coldest month (Tmin = BIO6), maximum temperature of the warmest month (Tmax = BIO5). For thermal variability profiles, we also extracted mean diurnal range (DR = BIO2) and temperature annual range (AR = BIO7). For rainfall regimes, we extracted precipitation of the wettest month (Prec wet = BIO13) and precipitation of the driest month (Prec dry = BIO14). Because amphibians are sensitive to hydric environments (Wells 2007; Hillman et al. 2009), we also extracted minimum potential evapotranspiration (PET) of the driest month, and maximum PET of the wettest month from CGIAR-CSI GeoPortal (Trabucco and Zomer 2010) (Fig. 2). We favored a combination of mean and extreme (e.g., coldest, warmest, driest, wettest) values since recent studies indicate climate extremes (e.g., warming or drought waves) may be key in natural selection of organismal physiological traits (Hoffmann and Sgrò 2011; Buckley and Huey 2016; Coleman and Wernberg 2020).

### Laboratory measurements of physiological traits

Given the questions asked in this paper, we have followed the set of experimental conditions summarized in the main manuscript but detailed in Supplementary Material 2. In the latter, we provide more details of all physiological data collection, animal acclimation, and manipulation, as well as decision-making for experimental setups.

One author (RPB) conducted all experiments, taking measurements only once for each trait per individual, in the following sequence: evaporative water loss (EWL) and water uptake (WU) within 3–5 days after field collection, and critical thermal minimum (CTmin) and maximum (CTmax) within, respectively, 4–6 and 6–8 days after field collection. Animals were returned to terraria immediately after measurements of EWL and WU and allowed to recover for one day before measuring CTmin. Similarly, after CTmin measurements, animals were returned to terraria and allowed to recover for 2 days before CTmax measurements. Before and after any experimental run, we checked animals for motor coordination, skin color, posture, and responsiveness. When individuals failed these checks or when they died, we did not consider them in the analysis. Missing data in the final dataset comprised 5%, 9.7%, 0.4%, and 15.5% for, respectively, EWL, WU, CTmin, and CTmax measurements (see Supplementary Material 3 and Table S1).

#### EWL rates

To measure water balance (EWL and WU), we followed previous standardized protocols (Bovo et al. 2016; Anderson et al. 2017; Gouveia et al. 2019); see details in Supplementary Material 2. Briefly, we standardized animals’ temperature and hydration state before the trials by holding each amphibian in individual PVC containers filled with 0.5 cm (smaller species) or 1 cm of water (larger species), placed inside a climate-controlled incubator (122FC model—Eletrolab) at 25°C for 1 h. Then, we measured EWL rates in a typical open-flow system at 25°C, containing a mass flow meter (SS-3 Subsampler, Sable Systems) supplying a stable airflow at 21.66 cm^3^ s^−1^ (1300 ml min^−1^), a RH/Dewpoint Controller (DG-4, Sable Systems) standardizing the incurrent relative humidity at 30% (water vapor density, WVD, saturated at 25°C = 23.09 g/m^3^), and a water vapor analyzer (RH-300 RH/Dewpoint Analyzer, Sable Systems) quantifying incurrent and excurrent air (empty chamber or containing the frog). All equipment was interfaced to a computer by an analog/digital unit (UI2–Sable Systems) to record changes in airflow and WVD every 1.0 s.

To quantify the EWL rates, we first calculated the WVD deficit, which refers to the difference between an empty container and one containing the animal (Spotila and Berman 1976). Then, total transepithelial EWL was corrected for unit area of exposed skin surface (2/3 of the total surface; McClanahan and Baldwin 1969) and expressed as μg_H2O_ cm**^−^**^2^ s**^−^**^1^. To minimize the animal's activity during EWL measurements, which could affect the records (e.g., Christian et al. 2017), we performed experiments during the day (opposite to their natural active period) in darkened chambers. To detect any significant changes in behavior and posture (Pough et al. 1983) that could affect the records, we often visually inspected individuals during trials. Data were discarded if an animal urinated during the experiment.

#### WU rates

To measure WU rates, immediately after the EWL trials in which animals usually lost 98–70% of their initial body masses (Supplementary Material 3), we placed each individual in containers (a Petri dish for smaller species: *D. minutus, P. cuvieri*; or a circular PVC container for larger species: *R. icterica, B. faber, L. latrans*) filled with tap water at a depth sufficient to cover their ventral region (Cree 1988). Animals were taken from the container and carefully blotted with paper tissue and weighed (±0.0001 g or 0.01 g) every 2 minutes for six consecutive times in a room at 25°C (Titon and Gomes 2015). We calculated WU from the linear regression between body mass increments against time. Then, using the estimated surface area in contact with water (1/3 of the total surface, McClanahan and Baldwin 1969), we calculated the rate of WU per unit area and expressed it as μg_H2O_ cm**^−^**^2^ s**^−^**^1^.

#### CTmin and CTmax

To assess thermal tolerances, we measured critical thermal minima (CTmin) and maxima (CTmax) in convective setups with constant cooling or heating ramp at a rate of 0.1°C/min (= 1°C/10 min = 6°C/h). We followed a meticulous protocol to measure core body temperatures, and also to prevent potential dehydration of animals during experiments (see Supplementary Material 2). Briefly, prior to experiments, we acclimated individuals in a climate-controlled chamber (EL101/2RS model—Eletrolab) at 25°C for 1 h. To measure internal body temperature (T_b_), we used digital thermometers of quick response (°C; ETI, EcoTemp Model) with an external probe (for the three large-bodied species), and a TC-1000 meter (Sable Systems) with a T-type thermocouple connected (for the two small-bodied species), which were inserted into the cloaca. To ensure the accuracy and repeatability of temperature readings, we periodically checked both thermometers placing both probes inside the climate-controlled chamber when we were not manipulating the animals. T_b_ readings were taken within 5–8 s after the loss of righting response.

### Index of thermal stress: WT

To estimate WT, we calculated the difference between CTmax and the maximum temperature of the environment (see Section ‘Study site and environmental variation descriptors’) associated with the geographical coordinates of the sampling sites (WT = CTmax – Tmax *sensu*; Duarte et al. 2012). This index indicates the average amount of environmental warming an organism can tolerate before temperatures become deleterious and ultimately lethal (Deutsch et al. 2008; Clusella-Trullas et al. 2021).

### Data analyses

We first tested for correlations between thermal and water balance traits within-species using individuals from different localities. Correlations between thermal tolerance and water balance traits were low to moderately correlated (Pearson r correlations ranging from –0.04 to 0.69, but only four moderate correlations across 42 total correlations across all species; Table S3). Thus, because variation in thermal tolerance was mostly independent from that of water balance traits, we analyzed these traits separately.

To test the relationships between within-species physiological traits (response variables) and altitude (continuous predictor variable), we used generalized linear models (GLM) with either Gaussian or Gamma distribution. We diagnosed model residuals using Bartlett's test for heteroscedasticity and Lilliefors’ test for normality. For thermal traits (CTmin, CTmax, Tbr) and index (WT), body mass was only included in the model when its effect was significant (see Supplementary Material 3). EWL and WU are mass-specific variables, so body mass is already accounted for. We analyzed the within-species trait variation separately for each mountain range because their relief characteristics, such as continentality, vegetation structure, age, and isolation of the biota (Körner 2007; Malhi et al. 2010), might be confounding variables. Although the climate of both mountain ranges is fairly similar (Fig. 2), other bioclimatic descriptors differed between mountain ranges (Fig. 2), thus supporting separate analyses. When we found a significant effect of altitude on any physiological trait, which was not the case for water balance traits (see Section ‘Water balance traits’ Results), we tested whether this effect was explained by bioclimatic variables known to determine climatic niche of amphibians (see Wiens et al. 2006; Gouveia et al. 2014). For this, we used linear models of physiological traits and bioclimatic variables as predictors. For CTmin, we chose the minimum temperature of the coldest month (Tmin = BIO6). For CTmax, we chose the maximum temperature of the warmest month (Tmax = BIO5). For Tbr, we chose temperature annual range (AR = BIO7), a proxy for seasonality. We did not test the influence of bioclimatic variables on WT because this index already includes Tmax of the environment.

Because microclimate may better represent the thermal environment surrounding the individuals, we also ran models using microclimatic data extracted from Kearney et al. (2014). We provide a detailed description of these methods in the Supplementary Material 2.

We determined sex based on external morphological characteristics or by field notes (e.g., males calling) but nor for all individuals. However, exploratory data analyses did not show differences in physiological traits between sexes (Supplementary Material 3). Therefore, we did not include sex as a predictor factor in the final models.

To estimate effect sizes, we calculated within-species trait differences between the lowest and highest altitudes in the same mountain range (Table 2). The variance-function-based coefficient of determination (adj R^2^) for Gamma GLMs (Zhang 2017) was calculated using the rsq R package (Zhang 2021). We did not quantify interspecific variation because it was not our study goal, and also because comparative analyses which adequately allow for control of phylogenetic relatedness generally require greater number of species (Blomberg et al. 2003; Revell 2010). All statistical analyses were conducted in R v.4.0.1 (R Core Team 2021).

**Table 2.** Results of generalized linear models testing the effects of altitude on physiological thermal tolerance (critical thermal minima, CTmin, and maxima, CTmax, and thermal breadth Tbr), on water balance (rates of evaporative water loss, EWL, and of water uptake, WU) traits, and on vulnerability to thermal stress (warming tolerance, WT) in five species of amphibians distributed in two mountain ranges (*Serra do Mar* and *Serra da Mantiqueira*) in the Brazil's Atlantic Forest.

**Serra do Mar**							
**CTmin**	Estimate	SD	*P*-value	R^2^ adjusted	Highest altitude (°C)	Lowest altitude (°C)	Whole species mean (°C)
Dmin	−0.0028	0.0001	**0.000**	0.35	3.6	7.9	6.1
Bfab	−0.0016	0.0003	**<0.001**	0.44	2.3	4.0	3.2
Rict	−0.0017	0.0006	**0.0070**	0.22	1.8	3.0	2.4
Pcuv	−0.0097	0.0035	**0.0113**	0.24	7.4	9.4	8.2
Llat	−0.0026	0.0005	**<0.001**	0.61	3.5	6.1	5.1
**CTmax**	Estimate	SD	*P*-value	R^2^ adjusted	Highest altitude (°C)	Lowest altitude (°C)	Whole species mean (°C)
Dmin	0.0000	0.0000	0.1975	0.01	31.1	33.4	33.3
Bfab	0.0002	0.0003	0.5869	0.00	38.2	38.4	38.5
Rict	−0.0015	0.0004	**0.0008**	0.36	38.3	39.4	38.8
Pcuv	0.0053	0.0059	0.3933	0.00	35.7	34.6	35.1
Llat	−0.0005	0.0002	**0.0468**	0.19	38.6	39.1	38.9
**Tbr**	Estimate	SD	*P*-value	R^2^ adjusted	Highest altitude (°C)	Lowest altitude (°C)	Whole species mean (°C)
Dmin	0.0013	0.0008	0.1139	0.04	26.9	25.6	26.5
Bfab	0.0017	0.0004	**0.0000**	0.48	35.9	34.4	35.3
Rict	0.0000	0.0007	0.9467	0.00	36.5	36.5	36.5
Pcuv	0.0152	0.0076	0.0735	0.21	28.4	25.4	26.9
Llat	0.0022	0.0005	**0.0003**	0.50	35.1	32.9	33.8
**WT**	Estimate	SD	*P*-value	R^2^ adjusted	Highest altitude (°C)	Lowest altitude (°C)	Whole species mean (°C)
Dmin	0.0043	0.0007	**0.0000**	0.55	7.3	3.0	6.5
Bfab	0.0061	0.0004	**0.0000**	0.90	13.4	8.0	11.6
Rict	0.0007	0.0004	0.0909	0.08	14.5	14.1	14.3
Pcuv	0.0078	0.0059	0.2194	0.06	10.9	9.3	10.1
Llat	0.0052	0.0002	**0.0000**	0.95	13.8	8.7	10.8
**EWL**	Estimate	SD	*P*-value	R^2^ adjusted	Highest altitude (μg cm^−2^ s^−1^)	Lowest altitude (μg cm^−2^ s^−1^)	Whole species mean (μg cm^−2^ s^−1^)
Dmin	0.0001	0.0001	0.5190	0.00	1.78	1.67	1.81
Bfab	−0.0001	0.0001	0.3810	0.00	1.11	1.34	1.33
Rict	−0.0003	0.0001	**0.0221**	0.15	1.50	1.87	1.65
Pcuv	−0.0001	0.0009	0.9275	0.00	2.68	2.70	2.69
Llat	−0.0001	0.0001	0.4967	0.00	1.81	1.95	1.85
**WU**	Estimate	SD	*P*-value	R^2^ adjusted	Highest altitude (μg cm^−2^ s^−1^)	Lowest altitude (μg cm^−2^ s^−1^)	Whole species mean (μg cm^−2^ s^−1^)
Dmin	−0.0182	0.0084	**0.0358**	0.07	59.43	83.76	64.79
Bfab	−0.0353	0.0173	0.0504	0.10	88.72	113.65	92.22
Rict	−0.0085	0.0194	0.6648	0.00	66.68	67.27	71.45
Pcuv	0.1794	0.0468	**0.0011**	0.41	69.82	33.58	54.29
Llat	0.0286	0.0233	0.2400	0.03	99.88	71.62	78.69
Dmin	–	–	–	–	6.1	–	6.1
Bfab	−0.0007	0.0003	0.0554	0.21	3.6	4.3	3.8
Rict	−0.0009	0.0004	**0.0281**	0.18	2.6	3.5	3.1
Pcuv	−0.0020	0.0009	**0.0446**	0.20	6.7	8.8	7.3
Llat	–	–	–	–	3.8	–	3.8
**CTmax**	Estimate	SD	*P*-value	R^2^ adjusted	Highest altitude (°C)	Lowest altitude (°C)	Whole species mean (°C)
Dmin	–	–	–	–	32.8	–	32.8
Bfab	0.0007	0.0005	0.2089	0.06	38.3	37.5	38.1
Rict	−0.0004	0.0003	0.1379	0.06	38.5	38.9	38.7
Pcuv	−0.0018	0.0004	**0.0020**	0.56	34.4	36.2	34.9
Llat	–	–	–	–	38.5	–	38.5
**Tbr**	Estimate	SD	*P*-value	R^2^ adjusted	Highest altitude (°C)	Lowest altitude (°C)	Whole species mean (°C)
Dmin	–	–	–	–	26.2	–	26.2
Bfab	0.0014	0.0006	**0.0477**	0.23	34.6	33.2	34.4
Rict	0.0005	0.0005	0.3546	0.00	35.8	35.3	35.6
Pcuv	0.0007	0.0007	0.3280	0.00	28.1	27.3	27.9
Llat	–	–	–	–	34.7	–	34.7
**WT**	Estimate	SD	*P*-value	R^2^ adjusted	Highest altitude (°C)	Lowest altitude (°C)	Whole species mean (°C)
Dmin	–		–	–	7.8	–	7.8
Bfab	0.0040	0.0005	**0.000**	0.81	13.2	9.0	12.3
Rict	0.0029	0.0003	**0.000**	0.84	13.5	10.4	11.9
Pcuv	0.0016	0.0004	**0.0043**	0.50	9.4	7.7	8.8
Llat	–	–	–	–	13.4	–	13.4
**EWL**	Estimate	SD	*P*-value	R^2^ adjusted	Highest altitude (μg cm^−2^ s^−1^)	Lowest altitude (μg cm^−2^ s^−1^)	Whole species mean (μg cm^−2^ s^−1^)
Dmin	–	–	–	–	2.01	–	2.01
Bfab	−0.0002	0.0002	0.3400	0.00	1.22	1.41	1.26
Rict	−0.0005	0.0001	**0.0012**	0.39	1.40	1.97	1.68
Pcuv	−0.0004	0.0002	0.0551	0.21	2.47	2.93	2.60
Llat	–	–	–	–	1.60	–	1.60
**WU**	Estimate	SD	*P*-value	R^2^ adjusted	Highest altitude (μg cm^−2^ s^−1^)	Lowest altitude (μg cm^−2^ s^−1^)	Whole species mean (μg cm^−2^ s^−1^)
Dmin	–	–	–	–	58.32	–	58.32
Bfab	−0.0485	0.0364	0.2190	0.08	83.01	133.95	98.29
Rict	0.0276	0.0121	**0.0346**	0.17	89.34	60.40	74.18
Pcuv	0.0135	0.0122	0.2860	0.02	53.70	39.49	49.91
Llat	–	–	–	–	85.17	–	85.17

Dmin: *Dendropsophus minutus*, Bfab: *Boana faber*, Rict: *Rhinella icterica*, Pcuv: *Physalaemus cuvieri*, Llat: *Leptodactylus latrans*. Dashes mean that specimens were not found in that site.

## Results

### Thermal and hydric environment along elevational gradients

Environmental thermal variables (Tmin, Tmean, and Tmax) decreased with altitude in both mountain ranges (Fig. 2). Daily and annual thermal range slightly increased with altitude (Fig. 2), ranging from, respectively, 9.3°C to 12.1°C and from 16.6°C to 21.4°C, for Serra do Mar and Serra da Mantiqueira. Precipitation and potential evapotranspiration (PET) did not change consistently with altitude (Fig. 2).

### Thermal traits

**Fig. 3:**
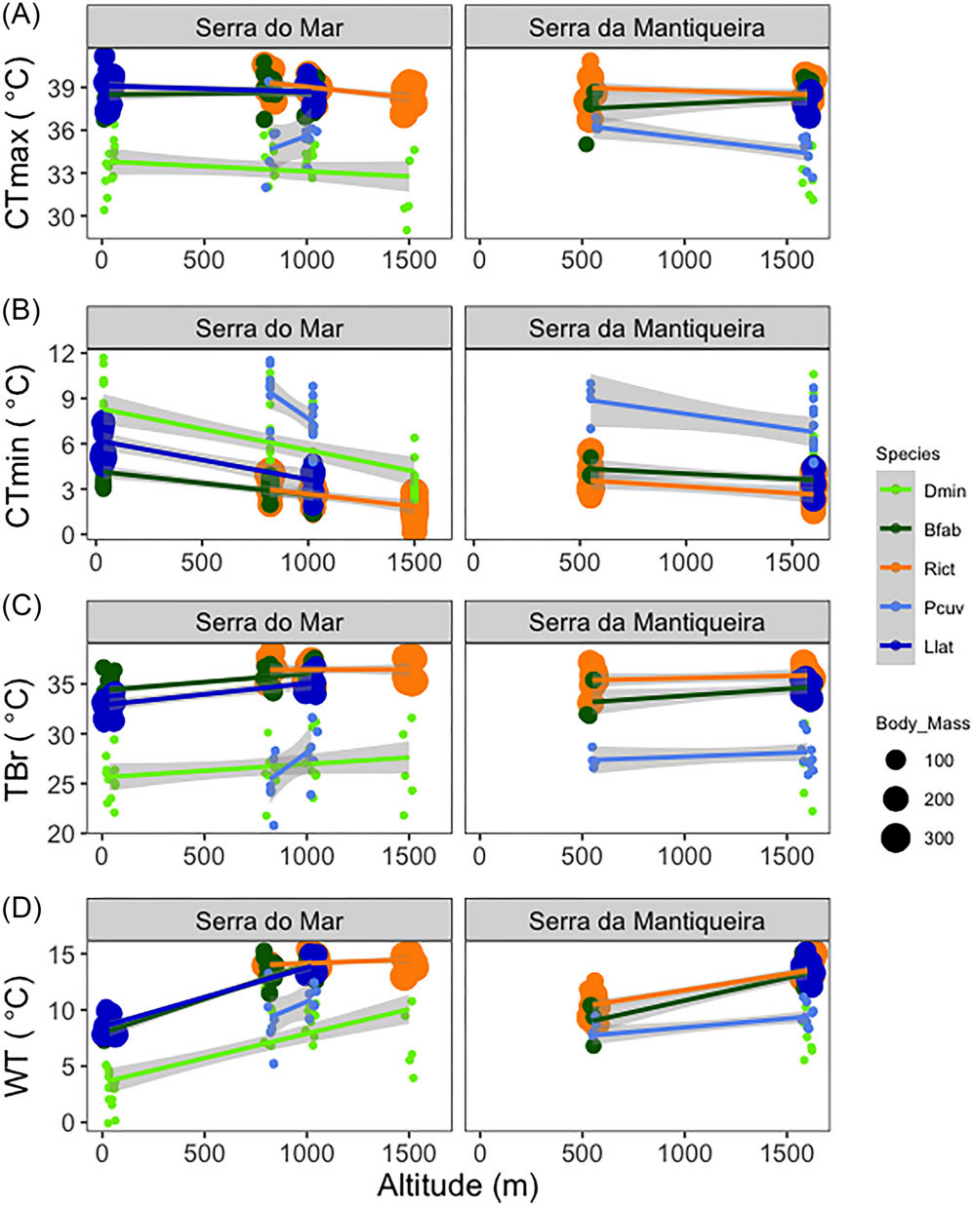
Relations between thermal traits and index with altitude. (A) Critical thermal maximum (CTmax) tolerance; (B) critical thermal minimum (CTmin) tolerance; (C) thermal breadth (Tbr); and (D) warming tolerance (WT) in five species of amphibians (Dmin: *Dendropsophus minutus*, Bfab: *Boana faber*, Rict: *Rhinella icterica*, Pcuv: *Physalaemus cuvieri*, Llat: *Leptodactylus latrans*) distributed along altitudinal gradients in two mountain ranges: *Serra do Mar* and *Serra da Mantiqueira*. Body mass in grams. CTmin within-species decreased consistently with altitude in all species, whereas CTmax decreased with altitude in three out of five species. Tbr increases with altitude in two species. WT increases with altitude in most species.

Within species thermal tolerance traits varied significantly with altitude, and cold tolerance changed more conspicuously with altitude than did heat tolerance. Altitude significantly explained from 18% to 61% of variation in CTmin in all species, whereas it explained less variation in CTmax in general, ranging from 19% to 36%, except for *P. cuvieri*, in which altitude explained 56% of variation in CTmax (Table 2). CTmin decreased with altitude in all species, and was 0.7°C to 4.3°C lower in highland populations when compared to their lowland counterparts (Fig. 3). The increase in cold tolerance with altitude was consistent between the two mountain ranges and independent of body mass. CTmax significantly decreased with altitude in three out of five species. In these three species, CTmax was 0.5°C to 1.8°C lower at highland populations than in lowland ones (Table 2, Fig. 3). CTmax was higher (∼38.5°C) in large body size species (*B. faber, R. icterica, L. latrans*) than in small ones (*D. minutus, P. cuvieri*; ∼34°C; Fig. 3).

The variation in thermal breadth (Tbr) explained by altitude was generally low (maximum of 50%; Table 2). Tbr was broader in highland than lowland populations in two out of five species, being 1.4°C to 2.2°C (Table 2). Tbr ranged from 25.4°C (*P. cuvieri* at Serra do Mar) to 36.5°C (*R. icterica* at Serra do Mar; Table 2, Fig. 3).

The variation in WT explained by altitude varied between 50% and 95% (Table 2). WT was greater in highland populations in most species, and was 1.7°C to 5.4°C higher than WT of lowland counterparts (Table 2). WT ranged from 3°C (*D. minutus* at Serra do Mar) to 14.5°C (*R. icterica* at Serra do Mar) (Table 2, Fig. 3). It is interesting to note that while *D. minutus* had the lowest WT, the population of this species at highest altitude showed among the greatest increases in WT.

### Bioclimatic variables as predictors of variation in thermal traits

As expected, all three bioclimatic variables had a significant effect on thermal trait variation. Variation in CTmin was significantly explained by Tmin (BIO6) in all species (20% to 61% of variation explained; Table 3, Fig. S2). The same three species (*R. icterica, P. cuvieri, L. latrans*,) in which we found a significant effect of altitude on CTmax also had CTmax variation significantly explained by Tmax (BIO5; 37% to 56% of variation explained; Table 3, Fig. S2). Finally, temperature annual range (BIO7) explained 23% to 50% of the variation in TBr in two species (Table 3, Fig. S2).

**Table 3.** Results of the Generalized Linear Models testing the relationship between thermal traits and specific bioclimatic variables for each of the five species of amphibians.

	**Serra do Mar**					**Serra da Mantiqueira**			
**CTmin ∼ Tmin**	Estimate	SD	*P*-value	R^2^ adjusted	**CTmin ∼ Tmin**	Estimate	SD	*P*-value	R^2^ adjusted
Dmin	0.062	0.013	**0.000**	0.23	Dmin	–	–	–	–
Bfab	0.190	0.038	**0.000**	0.43	Bfab	0.107	0.050	0.055	0.21
Rict	0.293	0.099	**0.006**	0.22	Rict	0.135	0.050	**0.028**	0.18
Pcuv	1.303	0.467	**0.011**	0.24	Pcuv	0.306	0.139	**0.045**	0.20
Llat	0.317	0.057	**0.000**	0.61	Llat	–	–	–	–
**CTmax ∼ Tmax**	Estimate	SD	*P*-value	R^2^ adjusted	**CTmax ∼ Tmax**	Estimate	SD	*P*-value	R^2^ adjusted
Dmin	0.002	0.003	0.668	0.00	Dmin	–	–	–	–
Bfab	−0.042	0.049	0.400	0.00	Bfab	−0.211	0.159	0.209	0.06
Rict	0.695	0.177	**0.001**	0.37	Rict	0.127	0.082	0.138	0.06
Pcuv	−2.133	2.391	0.393	0.00	Pcuv	0.529	0.132	**0.002**	0.56
Llat	0.083	0.037	**0.040**	0.17	Llat	–	–	–	–
**Tbr ∼ AR**	Estimate	SD	*P*-value	R^2^ adjusted	**Tbr ∼ AR**	Estimate	SD	*P*-value	R^2^ adjusted
Dmin	0.527	0.310	0.0982	0.05	Dmin	–	–	–	–
Bfab	0.665	0.136	**0.000**	0.43	Bfab	0.444	0.201	**0.048**	0.23
Rict	0.001	0.201	0.997	0.00	Rict	0.143	0.151	0.355	0.00
Pcuv	3.067	1.534	0.074	0.21	Pcuv	0.237	0.231	0.328	0.00
Llat	0.862	0.191	**0.000**	0.50	Llat	–	–	–	–

Critical thermal minima (CTmin), minimum temperature of the coldest month (Tmin = BIO6), critical thermal maxima (CTmax), maximum temperature of the warmest month (Tmax = BIO5), thermal breadth (Tbr), temperature annual range (AR = BIO7). Dmin: *Dendropsophus minutus*, Bfab: *Boana faber*, Rict: *Rhinella icterica*, Pcuv: *Physalaemus cuvieri*, Llat: *Leptodactylus latrans.* Dashes mean that specimens were not found in that site.

Using microclimate data, we found that variation in CTmin was significantly explained by minimum temperatures (tmin) in four out of five species (18% to 61% of the variation explained; Table S4). However, we found inconsistent patterns of change in CTmin, some of them increasing (negative slopes) or decreasing with tmin in some species. Also, the pattern for CTmax did not improve comparing to macroclimate (Table 3), with CTmax increasing or decreasing (negative slopes) with maximum temperatures (tmax) (Table S4). Finally, we found a similar pattern of change in Tbr explained by annual temperature range (ar = tmax – tmin) as for macroclimatic models. Microclimate data are outputs of algorithms applied to macroclimate data (Kearney et al. 2014), and may not represent accurately microclimate of our specific studies sites (e.g., South America and altitudinal regions). Therefore, given that microclimate data did not generally explain more variation in thermal tolerance than macroclimate did, we focus our discussion only in macroclimatic information.

### Water balance traits

**Fig. 4:**
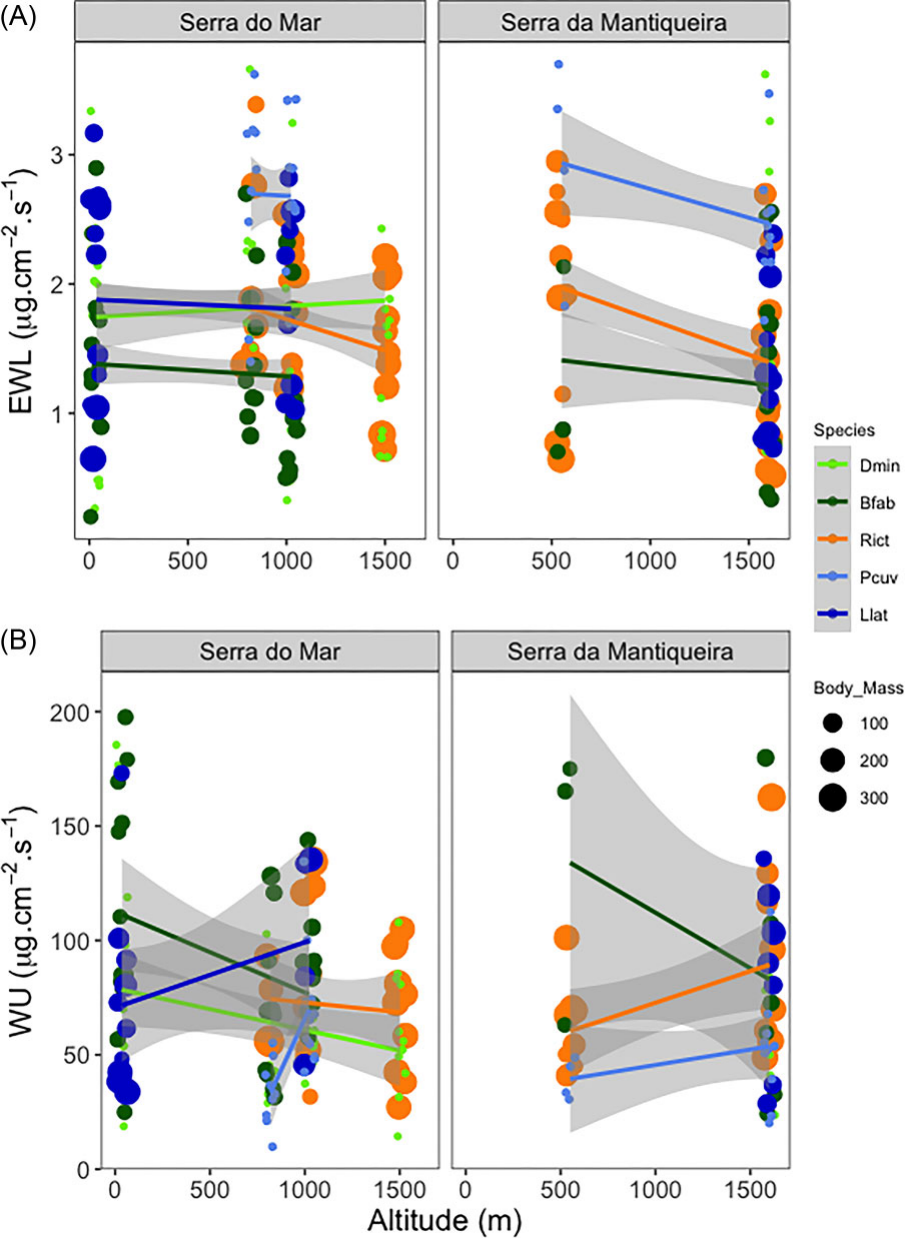
Relations between water balance traits altitude. (A) Rates of evaporative water loss (EWL) and (B) water uptake (WU) in five species of amphibians (Dmin: *Dendropsophus minutus*, Bfab: *Boana faber*, Rict: *Rhinella icterica*, Pcuv: *Physalaemus cuvieri*, Llat: *Leptodactylus latrans*) along altitudinal gradients in two mountain ranges: *Serra do Mar* and *Serra da Mantiqueira*. Body mass in grams. Rates of water loss and gain did not change consistently along altitudinal gradients.

In contrast to thermal tolerance traits, water balance traits did not show consistent variation with altitude. Rates of EWL remained unchanged with altitude in most species. Only *R. icterica* showed EWL lower in highlands than in lowlands (Table 2, Fig. 4). Rates of WU changed in only a few cases with altitude across species. WU decreased by 30% (*D. minutus* at Serra do Mar) or increased by 47% (*R. icterica* at Serra da Mantiqueira) and 107% (*P. cuvieri* at Serra do Mar) with altitude (Table 2, Fig. 4). Given that altitude did not have a consistent effect on water balance traits, we did not run any further linear models with bioclimatic variables as predictors.

## Discussion

According to JH, we would expect tropical species to have limited distributions along elevational gradients because physiological specialization should act as a dispersal barrier, limiting gene flow (Janzen 1967; Ghalambor et al. 2006). However, we must go beyond JH to understand how tropical species can tolerate climate variation found in broad distributional ranges. In this work, we tested hypotheses underlying changes in thermal breadth and limits, and also in water balance responses in populations of tropical species of amphibians distributed along elevational gradients. While our results partially agreed with one component of the JH, i.e., broader thermal variation leading to broader thermal tolerance breadths (the CVH) in only two out of five species, we found stronger support for CTmin changing more than CTmax (the cold-variability and heat-invariant hypotheses) with altitude. This indicates that changes in thermal limits did not always imply changes in thermal tolerance breadths. We also found that patterns of water balance did not show consistent variation with altitude, as well as low correlation between hydric and thermal traits.

These mismatches between variation in thermal breadth and limits, and water balance, emerged due to populational responses to climate variation along elevational gradients, which were context-dependent and species-specific, and also associated with the spatial variation of temperature and water along altitude. Below we discuss in detail these responses and their broad implications.

### Thermal and water balance traits show divergent patterns to increasing altitude

Our findings for thermal traits corroborate the cold-variability and heat-invariant hypotheses because CTmin decreased consistently with altitude in all species, but CTmax did not drop consistently. While some studies have reported significant changes in CTmax with altitude in amphibians (Brattstrom 1968; [Bibr bib53][Bibr bib53]; Catenazzi et al. 2014; Pintanel et al. 2019), others did not (Heatwole et al. 1965; Brattstrom 1968; Christian et al. 1988). A general explanation for this incongruence may depend on the behavior buffering against selective pressures (Bogert 1949; Huey et al. 2003; Muñoz 2022), given that frogs are nocturnal animals that likely avoid exposure to maximum temperatures at daytime and, as consequence, populations may not be under selective pressures related to high temperatures. Indeed, CTmax was not strongly related to maximum environmental temperatures (Tmax; Table 3, Fig. S2), corroborating the idea of no or low selective pressure of Tmax on CTmax. Conversely, CTmin was consistently related to minimum environmental temperatures in all our species (Table 3, Fig. S2), suggesting that the lowest temperatures drive selection for cold tolerance (Sunday et al. 2011; Buckley and Huey 2016). CTmax may also be more constrained to change (Hoffmann et al. 2013), which would result in lower evolutionary rates compared with CTmin (Araújo et al. 2013; Sunday et al. 2014). While some recent studies with tropical amphibians in the Andean mountains report a higher evolutionary rate for CTmin compared to CTmax (von May et al. 2019), others did not (Pintanel et al. 2019). Hence, it seems plausible that other factors may influence the evolution of CTmin and CTmax, such as differences in selection strength (e.g., Kingsolver et al. 2012), in the degree of functional constraints of thermal tolerance with other physiological traits (e.g., Pörtner 2002; Jackson 2007) or variation in ecological factors, such as microhabitat selection and body size (von May et al. 2019). In this regard, our results suggest that body size may play a role in the variation in thermal tolerances among species. Accordingly, CTmax of small body size species (*D. minutus, P. cuvieri*) was ca. 5°C lower than in large species (*H. faber, R. icterica, L. latrans*), while CTmin did not show a clear pattern of variation with body size.

Although we found significant responses of thermal traits to altitude, we acknowledge that we did not sample all species at all elevations, including the lowest elevation for some species. While this caveat may have underestimated the physiological change for some species, the direction of the relationships (trait and altitude) is mostly consistent across species, independent of how many locations were sampled (see Table S1 for population sample sizes). Therefore, we believe we captured the general pattern of intraspecific variation in physiological tolerances across altitude in the studied species.

In contrast to the thermal tolerance responses, water balance traits showed no clear pattern of change with altitude. This result might be a consequence of the thermal environment changing more drastically with elevation on the studied mountains than the hydric environment (Fig. 2). Consequently, the relative stability in the hydric environment along altitude, at least for the mountains we studied that show moderate altitude when compared with highlands worldwide (e.g., Andes, Tibet), may have prevented selection to shift water loss or gain in the populations we sampled. In fact, it remains unknown which magnitude of change in the hydric attributes of the environment would alter physiological traits associated with water balance in tropical anurans. Nevertheless, variation in water balance traits sometimes correlate with variation in the environment. Studies with tropical amphibians found that water balance traits covary with large-scale gradients of aridity (e.g., Van Berkum et al. 1982; Gouveia et al. 2019). For instance, some amphibians may either evolutionarily increase skin resistance to EWL or body size, both as a mechanistic response of water economy to cope with more arid environments (Gouveia et al. 2019). Given our increasing knowledge on the diversity of patterns and mechanisms involved in water balance responses to climate variation, further studies—especially with tropical taxa—are needed to assess whether and how water balance traits might have evolved, especially in response to large-scale ecological and evolutionary contexts (e.g., Moen et al. 2022).

### Mixed support for CVH

Thermal breadth is commonly used as a proxy to infer species’ potential to respond to changes in temperature (Angilletta 2009). As expected, we found that highland populations have broader Tbr by 1.4 to 2.2°C, but only in two out of the five species studied. Broader Tbr were possibly driven by the greater variation in annual temperature range in upland areas. Interestingly, broader Tbr could be merely a by-product of a consistent decrease in CTmin with elevation but by a smaller shift in CTmax (Araújo et al. 2013; Sunday et al. 2014). However, while CTmin changed more than CTmax, consistent decreases in CTmin did not always result in increases in Tbr. This emphasizes the crucial importance of measuring intraspecific variation in both upper and lower thermal tolerances to better understand potential capacity of tropical species to respond to climate changes (e.g., Herrando-Pérez et al. 2019). Indeed, the more we generate data on tropical systems, more nuanced the responses to climate changes seems to be (e.g., von May et al. 2019). For instance, CTmin and CTmax of several tropical species of amphibians (Catenazzi et al. 2014; von May et al. 2017, 2019; Pintanel et al. 2019; Reider et al. 2021, our study) are comparable to those of temperate taxa (Fig. S3). This suggests that thermal tolerances of tropical species may have been underestimated, raising questions about whether many of these species would be highly vulnerable to climate stress.

### Differential vulnerability to thermal stress

We found that WT was higher in highland populations mostly because maximum environmental temperatures (Tmax *sensu*Duarte et al. 2012) decreased more with altitude than CTmax. Given that WT is an index commonly used to address vulnerability to thermal stress (Deutsch et al. 2008), we may infer that highland populations should be less vulnerable to thermal stress than their lowland counterparts, as well as less vulnerable to global warming. However, a thought-provoking finding is that the vulnerability of our tropical lowland frog populations was also low because local climate characteristics—particularly Tmax—were far below their thermal physiological limits (up to 14.5°C; Fig. 3). Thus, not all tropical forest-dependent species may be prone to suffer from thermal stress by warming predicted by IPCC (2014), even in worst-case scenarios of air temperature increases (4–4.5°C). This outcome was unexpected because most tropical species are thought to be vulnerable to global warming (e.g., Tewksbury et al. 2008). One noteworthy exception in our study is the small-bodied tree frog, *D. minutus*, in which the lowland population (35 m a.s.l.) is in danger of not being able to cope with an increase of 4°C, given that its WT is 3°C. Yet, this species also exemplifies how climbing the mountain can alleviate thermal stress (e.g., Sinervo et al. 2018), since WT of highland populations is up to 4.3 °C higher than in lowlands.

These findings highlight the diversity of responses to thermal changes in tropical species in which interactions between local temperature characteristics and physiology may lead to differential vulnerability to thermal stress. This is supported by other studies with tropical amphibians, in which vulnerability to thermal stress varied with altitude but also with natural history traits (von May et al. 2019) or varied between open and forest habitats (Pintanel et al. 2019). Thus, vulnerability to thermal stress also seems context dependent (Buckley and Kingsolver 2021), emphasizing that sampling individuals from just a single locality to represent the entire species may under or overestimate its vulnerability (Herrando-Pérez et al. 2019; Senior et al. 2019). This is especially relevant for species with broad distributions that may encompass environmental gradients.

### Conclusions

By adopting an intraspecific approach to test hypotheses underlying changes in thermal breadth and limits, and also water balance responses to climatic variation along altitude, our findings indicate that tropical frog and toad species that can disperse along the mountains do so by mainly changing their cold tolerance and, in a lesser degree, heat tolerance. Moreover, we found a mismatch between changes in thermal breadth and limits, which adds an extra layer of difficulty to predict species responses to climate variation across environmental gradients in the tropics. In addition, water balance traits do not seem to limit the species distributions across elevation. Taken together, we found heterogeneous populational responses among physiological traits along altitude, but also among species that show differences in the magnitude of responses to climatic variation across elevations. This diversity in hydrothermal responses may be driven by other factors than just environmental variation, such as behavior (Muñoz 2022), energy balance (Riddell et al. 2018), species life history traits (e.g., body size, microhabitat selection; von May et al. 2019), and/or ontogeny, all factors that drive physiological traits and limits to be dynamic concepts rather than fixed values for a species (e.g., Bovo et al. 2018; Navas et al. 2022). Our results also showed the relevance of considering spatial variation in climate when addressing organismal responses (Sears et al. 2016, 2019), shown by the differential but low vulnerability to thermal stress in both lowland and highland populations. This, in turn, brings generalizations about high vulnerability of many tropical taxa into question (e.g., Walters et al. 2012; von May et al. 2019), especially lowland forest inhabitants that would be living close to their CTmax (Tewksbury et al. 2008; Huey et al. 2009; Sunday et al. 2014).

In summary, several responses to climatic variation in tropical species may not conform to predictions made by either the CVH or other important hypotheses concerning physiological variation (e.g., Bozinovic et al. 2011). This is critical because several meta-analyses assessing vulnerability to climate changes, as well as many ecogeographical rules, rely on physiological information which currently may not represent the full range of responses in tropical species. In a broad perspective, we need more empirical physiological data on tropical systems to overcome geographical bias when predicting climate change impacts on biodiversity (White et al. 2021; Herrando-Pérez et al. 2023).

## Supplementary Material

obad009_Supplemental_Files

## Data Availability

Data available at github.com/rpbovo/22_physiology_altitude_IOB. R code is provided as Supplemental Material 3. DNA sequences are available in GenBank (Acc. Num. OL342233–OL342295).
