# Supplementary material for: Beyond Janzen's Hypothesis: How Amphibians That Climb Tropical Mountains Respond to Climate Variation
Source: Integr Org Biol. 2023 May 3;5(1):obad009. doi: 10.1093/iob/obad009 (PMC10155226; doi:10.1093/iob/obad009)
Supplement: obad009_Supplemental_Files [file obad009_supplemental_files.zip › Supplementary_Material1_4tables_3figures_IOB_.docx]

**Supplementary material (1)**

**(1) 4 tables, 3 figures**

**Table S1.** Number of individuals, range of body masses, and number and percentage of deaths after critical thermal maxima (CTmax) trials for the five study species of amphibians. No individual died during EWL trials, but one died during CTmin experiments (data not considered in data analysis, see Methods). Missing data in the final dataset comprised 5%, 9.7%, 0.4% and 15.5% for, respectively, rates of evaporative water loss (EWL) and water uptake (WU), CTmin, and CTmax measurements (see Supplementary Material 3). Water balance data (EWL, WU) not included in data analyses were from individuals removed because of unreliable measurements (see Supplementary Material 2).

**Table S2.** GenBank accession numbers for individuals included in the genetic analyses, along with data on the sampling localities. CFBH represents nucleotide sequences from species available at the CFBH herpetological collection, while KJ and MT are sequences extracted from GenBank (See Figure S1 for more details).

**Table S2.** Continuation.

**Table S3.** Pairwise correlation within-species between thermal tolerance (critical thermal minima, CTMin, and maxima, CTMax) and water balance (rates of evaporative water loss, EWL, and of water uptake, WU) traits in five species of amphibians (Dmin: *Dendropsophus minutus,* Bfab: *Boana faber,* Rict: *Rhinella icterica*, Pcuv: *Physalaemus cuvieri*, Llat: *Leptodactylus latrans*) studied at the Atlantic Forest of Brazil (see study locations in Figure 1).

**Table S4**. Results of the Generalized Linear Models testing the relationship between thermal traits and specific **micro**climatic variables for each of the five species of amphibians. Critical thermal minima (CTmin), minimum temperature of the coldest month (tmin), critical thermal maxima (CTmax), maximum temperature of the warmest month (tmax), thermal breadth (Tbr), temperature annual range (ar). Dmin: *Dendropsophus minutus,* Bfab: *Boana faber,* Rict: *Rhinella icterica*, Pcuv: *Physalaemus cuvieri*, Llat: *Leptodactylus latrans.* Dashes mean that specimens were not found in that site or that the model could not be ran (see Supplementary Material 2).

**Figure S1.** Intraspecific genetic structure within five species of amphibians showing differences between lowland (red dots) and highland (blue dots) populations in two mountain ranges. Phylogenies were inferred using 16S mtDNA under Maximum Likelihood (ML), and only nodes with ML bootstrap support higher than 95 % are included in the figure. Scale bars represent branch length. Specimen codes starting with RPB represent those we collected in the field. CFBH represents nucleotide sequences from species available at the CFBH herpetological collection, while KJ and MT are sequences extracted from GenBank (See Table S2 for more details).

**Figure S2.** Relationships between thermal traits and specific bioclimatic variables for each of the five species of amphibians. Critical thermal minima (CTmin), minimum temperature of the coldest month (Tmin = BIO6), critical thermal maxima (CTmax), maximum temperature of the warmest month (Tmax = BIO5), thermal breadth (Tbr), temperature annual range (AR = BIO7). Dmin: *Dendropsophus minutus,* Bfab: *Boana faber,* Rict: *Rhinella icterica*, Pcuv: *Physalaemus cuvieri*, Llat: *Leptodactylus latrans* (see Table 3 for more details on the results of the Generalized Linear Models testing the relationships).

**Figure S3.** Thermal tolerances of tropical (triangles) and temperate (circles) adult amphibians across latitudes. Sources: GlobTherm (Bennett et al. 2018) is the more comprehensive compilation of thermal tolerances of organisms; we only included data for Amphibia (Anura and Caudata). We also included data from the present study (Brazil’s Atlantic Forest) and others (from the Ecuadorian and the Peruvian Andes mountains and lowlands in South America) not included in the GlobTherm: Catenazzi et al. 2014, von May et al. 2017, von May et al. 2019, Pintanel et al. 2019, Reider et al. 2021.

**References**

Bennett JM, Calosi P, Clusella-Trullas S, Martínez B, Sunday J, Algar AC, Araújo MB, Hawkins BA, Keith S, Kühn I. 2018. GlobTherm, a global database on thermal tolerances for aquatic and terrestrial organisms. Sci Data 5:1–7.

Catenazzi A, Lehr E, Vredenburg VT. 2014. Thermal physiology, disease, and amphibian declines on the eastern slopes of the Andes. Conserv Biol 28:509–17.

Pintanel P, Tejedo M, Ron SR, Llorente GA, Merino-Viteri A. 2019. Elevational and microclimatic drivers of thermal tolerance in Andean *Pristimantis* frogs. J Biogeogr 46:1664–75.

Reider KE, Larson DJ, Barnes BM, Donnelly MA. 2021. Thermal adaptations to extreme freeze–thaw cycles in the high tropical Andes. Biotropica 53:296–306.

von May R, Catenazzi A, Corl A, Santa-Cruz R, Carnaval AC, Moritz C. 2017. Divergence of thermal physiological traits in terrestrial breeding frogs along a tropical elevational gradient. Ecol Evol 7:3257–67.

von May R, Catenazzi A, Santa-Cruz R, Gutierrez AS, Moritz C, Rabosky DL. 2019. Thermal physiological traits in tropical lowland amphibians: Vulnerability to climate warming and cooling. PLoS One 14:e0219759.
