# Supplementary material for: Beyond Janzen's Hypothesis: How Amphibians That Climb Tropical Mountains Respond to Climate Variation
Source: Integr Org Biol. 2023 May 3;5(1):obad009. doi: 10.1093/iob/obad009 (PMC10155226; doi:10.1093/iob/obad009)
Supplement: obad009_Supplemental_Files [file obad009_supplemental_files.zip › Supplementary_Material2_methods_IOB_.docx]

**Supplementary Material (2)**

***Evaporative Water Loss (EWL) measurements***

***Water Uptake (WU) measurements***

***Phylogeographic structure among populations***

***Linear models with microclimatic data***

***References***

**(2) Supplementary methods**

Given the questions asked in this paper, we have followed the set of experimental conditions summarized in the main manuscript but also detailed in this supplementary material. Herein, we provide details of all physiological data collection, animal acclimation, and manipulation, as well as decision-making for experimental setups.

It is worth to note that we are aware that virtually all physiological ecology parameters are to some extent method dependent. A direct consequence is that our approach informs about the physiological state of animals soon after capture and before any compensatory responses may occur. Another consequence is that we did not assume that a given population or species has a fixed value of CTmin, CTmax, EWL, or WU. In fact, the diversity in hydrothermal responses may be driven by several factors (see Section “Conclusions” in the main manuscript) that partially or fully influence physiological limits as dynamic concepts more than fixed values for a species (e.g., Bovo et al. 2018, Navas et al. 2022).

***Evaporative Water Loss (EWL) measurements***

We acclimated individuals inside a climate-controlled incubator (122FC model - Eletrolab) at 25 °C for 1 h. We quantified rates of EWL at 25 °C for the following reasons. First, average temperatures in the Brazilian Atlantic Forest are between 22 and 25 °C (Colombo and Joly 2010). Second, since many of our study sites were at highlands, resulting in mean environmental temperatures closer to 22 °C than 25 °C (Figure S1), we favored 25 °C because it is predicted an increase of 3 °C in the mean temperature in the Atlantic Forest by the end of the 21^st^ century (IPCC 2014). Finally, we did not measure EWL under different combinations of temperature and/or relative humidity (UR), because this approach would extend measurements for several days, which could trigger acclimatory responses that would bias our estimates of thermal tolerance. To ensure that animals were fully hydrated at the beginning of the experiment to measure EWL, we held each amphibian in individual PVC containers filled with 0.5 cm (smaller species) or 1 cm of water (larger species), placed inside a climate-controlled incubator (122FC model - Eletrolab) at 25 °C for 1 h.

Following the standardization of temperature and hydration state, each individual was carefully blotted with paper tissue, its urinary bladder was emptied by gently pressing the abdomen, and then we recorded body mass (to the nearest 0.0001 g or 0.01 g). Next, we placed each individual in a dry circular PVC container (8 cm in diameter) and returned it to the incubator to start the EWL measurements. The container operated as an automated open flow system, with two connections that served for the incurrent and excurrent airflows. Before reaching the animal´s container, airflow was directed to a RH/Dewpoint Controller (DG-4, Sable Systems) to standardize the incurrent relative humidity at 30% (water vapor density, WVD, saturated at 25 °C = 23.09 g/m^3^). While RH at 30% could be considered relatively low when taking into account the typically high humid environments in the warm/wet season in the Atlantic Forest (Oliveira-Filho and Fontes 2000, Colombo and Joly 2010), we judged this experimental scenario to best mimic natural conditions than dry air (~1% of RH) that is often used as experimental baselines in several studies assessing rates of EWL. Consequently, we quantified appropriate water balance responses of amphibians under a particular scenario of low RH, but plausible to the conditions found in study sites under 25 °C.

The incurrent airflow was generated by a pump, combined with a mass flow meter (SS-3 Subsampler, Sable Systems), supplying a stable airflow at 21.66 cm^3^.s^-1^ (1,300 ml.min^-1^) and providing a 99% of air turnover in the container with the animal every 3.5 min (Lasiewski et al. 1966). With this, we minimized the chance of artificially reducing the rates of EWL due to increasing air humidity inside the container, since low airflows associated with typical high rates of EWL in amphibians may underestimate the actual rates under the given conditions.

The excurrent airflow was continuously monitored by a water vapor analyzer (RH-300 RH/Dewpoint Analyzer, Sable Systems), which was interfaced to a computer by an analog/digital unit (UI2 – Sable Systems) to record changes in WVD every 1.0 second. To analyze the recorded data, we used the Expedata software (Sable Systems). Typically, measurements lasted for 30 minutes (small species) or 1 hour (large species) per individual. For quantitative estimates, we selected a steady-state period of readings lasting for at least 10 minutes, usually during the last half of the measurement period. To check the integrity of the system, we always measured an empty container (to return to 30% RH) before and after each individual measurement.

To estimate the rates of EWL, we first calculated the WVD deficit, i.e. the difference between an empty container and one containing the animal, thus the increment in water content in the system (Spotila and Berman 1976). Then, total transepithelial EWL was corrected for unit area of exposed skin surface (2/3 of the total surface) and expressed as μg_H2O_.cm**^−^**^2^.s**^−^**^1^. We estimated the exposed surface area of individual amphibians on the basis of their body mass (McClanahan Jr and Baldwin 1969, Young et al. 2005). We assumed EWL through the respiratory system to be negligible (Spotila and Berman 1976, Bentley and Yorio 1979, Wygoda 1984) and did not consider it in our EWL estimates.

To minimize the activity of the animals during EWL measurements, which could affect the records (e.g., Christian et al. 2017, Riddell and Sears 2017), all determinations were performed during the day (opposite to their natural active period) in darkened chambers. In addition, we often visually inspected individuals during trials for their posture (important for accurate estimation of body surface area) to detect any significant changes in behavior that could affect the records. We did not observe abnormal behaviors for any individual before, during, or after EWL measurements. The data was discarded if an animal urinated during the experiment.

***Water Uptake (WU) measurements***

To measure WU rates, immediately after the EWL trials, in which animals usually lost 98-70 % of their initial body masses (Supplementary Material 3), we placed each individual in containers (a Petri dish for smaller species: *Dendropsophus minutus*, *Physalaemus cuvieri*; or a circular PVC container for larger species: *Rhinella icterica*, *Boana faber*, *Leptodactylus latrans*) filled with water at a depth sufficient to cover their ventral region (Cree 1988). Animals were taken from the container and carefully blotted with paper tissue and weighed (± 0.0001 g or 0.01 g) every 2 minutes for six consecutive times in a room at 25 °C (Titon and Gomes 2015). We calculated WU from the linear regression between body mass increments against time. Then, using the estimated surface area in contact with water (1/3 of the total surface, McClanahan Jr and Baldwin 1969, Young et al. 2005), we calculated the rate of WU per unit area and expressed it as μg_H2O_.cm**^−^**^2^.s**^−^**^1^.

***Thermal tolerance: CTMin and CTMax measurements***

To assess thermal tolerances, we measured the critical thermal minima (CTMin) and maxima (CTMax). Both critical temperatures have been widely used to provide information about thermal limits to activity in ectotherms (Lutterschmidt and Hutchison 1997, Hutchison and Dupré 1992). These critical temperatures often result in the loss of motor/righting response (Cowles and Bogert 2006) and have been largely used in establishing the limits of thermal tolerance in amphibians (Brattstrom 1963, 1968, Seibel 1970, Claussen 1973, Miller and Packard 1977, Christian et al. 1988, Navas et al. 2007, Sanabria et al. 2012, Catenazzi et al. 2014, Pintanel et al. 2019). However, thermal tolerance estimates may vary depending on methodology. One of the main causes is that numerous studies use ecologically unrealistic abrupt changes (1 °C/min) in heating/cooling temperature (e.g., Terblanche et al. 2007, Rezende et al. 2011). Here, we pondered all factors, including phylogeny, ecology, morphology, and physiology of our focal species, as well as the study purpose. Therefore, we consider our standardization for measuring ecological critical limits in convective conditions suitable for both CTMin and CTMax estimates.

Prior to experiments, we held animals in individual thin polycarbonate containers (5 and 9 cm of diameter for, respectively, small-bodied species [*D. minutus*, *P. cuvieri*] and large-bodied ones [*R. icterica*, *B. faber*, *L. latrans*]) covered with nylon net (to ensure rapid thermal equilibrium of the recipient with the chamber temperature) placed inside a climate-controlled chamber (EL101/2RS model – Eletrolab) at 25 °C for 1 h. We observed in previous experiments that not all individuals started the experiments at the exact temperature of the chamber (25 °C), due to the typically high permeable amphibian skin, entailing transepithelial evaporation that slightly reduced body temperature. However, this procedure allowed us to have the best acclimation possible for all species. Thus, animals started the experiments under comparable conditions under the air/terrestrial conditions. To prevent acute dehydration stress during thermal tolerance experiments (which lasted for 3h to 4h), we placed a thin piece (4 cm x 3 cm) of moist paper tissue on the bottom of containers, but animals did not prefer to stand on it. Therefore, there is no evidence that it affected thermal tolerance estimates, causing biased patterns among populations or species.

Then, we programed the software Sitrad (version 4.12, Full Gauge Controls) to produce a constant cooling or heating ramp at a rate of 0.1 °C/min (= 1 °C/10 min = 6 °C/h). We chose these cooling/heating rates instead of more acute changes because: (i) as we noticed in preliminary trials, the loss of the righting response in our focal species was much more difficult to be identified under faster rates of temperature (e.g., 0.7 °C /min) change instead of slower ones (0.1 °C /min); (ii) slower ramping essays may be more ecologically relevant in assessing thermal tolerances when compared to more acute trials (Terblanche et al. 2007, Rezende et al. 2011, Terblanche et al. 2011); and (iii) the chosen rate would favor our thermal indices of vulnerability (calculated with time-series changes in air temperatures) to be more realistic when compared to, for instance, rates such as 1 °C/min (as performed in many studies of thermal tolerances with tadpoles and adult amphibians with small body sizes).

To measure internal body temperature (T_b_), we used digital thermometers of quick response (^o^C; ETI, EcoTemp Model) with an external probe (for the three large-bodied species), and a TC-1000 meter (Sable Systems) with a T-type thermocouple connected (for the two small-bodied species), which were inserted into the gastrointestinal tract via the cloaca. Both thermometers were periodically checked to ensure equality in measurements. We always placed both probes inside the climate-controlled chamber when we were not manipulating the animals to confirm the repeatability of temperature readings. We used two apparatuses to measure T_b_’s to (i) avoid any injury to the animals, due to large differences in body sizes, and also (ii) to ensure the most reliable T_b_’s records, since none of the probes showed adequacy (stiffness) to be inserted into the cloacae of both small- and large-bodied animals. This had no impact in our results, since our main goal was not to compare among-species thermal tolerances*.*

To manipulate animals, we always used surgical gloves to reduce heat transfer from the experimenter hand to the animals body. T_b_ readings were taken within 5-8 seconds after the loss of righting response. Individuals that did not recover after the experiment were not considered in the analysis. This was the case for some individuals during CTmax trials (Table S1).

***Phylogeographic structure among populations***

To ensure that the species were broad distributed along the elevational gradients, and not potential cryptic species, we performed a genetic analysis using the mitochondrial 16S rRNA gene to quantify the phylogeographic structure within species across altitude (Table S2). First, we extracted total DNA from one to four individuals per species and locality (Maniatis et al. 1982). We were unable to obtain genetic data from a few samples, but complemented our data set by including available tissue samples from the same species and localities (Célio F. B. Haddad Amphibian Collection) or from GenBank (Table S2). We used only a single mitochondrial gene (16S) to perform the analysis of gene flow between lowland and highland populations. Although using more genes would give a better resolution, mitochondrial genes evolve faster than nuclear ones (Galtier et al. 2009, Medina et al. 2021), potentially revealing recent genetic differentiation, which would support local adaptation within-species. Moreover, most studies usually describe patterns of co-variation between traits and the environment, but rarely evaluate whether observed trait variation is due to local adaptation or phenotypic plasticity (Bovo et al. 2018), as we have done here even if using a single gene.

Then, we amplified one fragment of the mitochondrial 16S rRNA gene (16S) using primers 16Sar-L and 16Sbr-H (Kessing et al. 1989), following cycling conditions described in Lyra et al. (2017). After enzymatically purifying PCR products, we sent products to Macrogen Inc. (Seoul; South Korea) for sanger sequencing. We checked chromatograms for quality and then trimmed them using Geneiuos R11 (Kearse et al. 2012). We aligned all sequences using MUSCLE (Edgar 2004) and inferred a Maximum Likelihood Tree in MEGA v.7 (Kumar et al. 2016) to describe within-species genetic structure. To calculate branch support of the species tree, we used 1,000 bootstrap samples to reconstruct the tree with each sample. We show only branches with 95% or higher support.

***Linear models with microclimatic data***

We extracted microclimatic data for air temperature at 1 cm above soil substrates at 50% shade from Kearney et al. (2014; https://figshare.com/collections/microclim_Global_estimates_

of_hourly_microclimate_based_on_long_term_monthly_climate_averages/878253). These data are in the netCDF format and needed to be manipulated before entering them in the models. Each file provides estimates of air temperature at 1 cm above soil at 50% shade for each month and for each hour of the day for the whole globe. We first extracted temperature data for each locality sampled. Then, we averaged the temperature for each month across all hours for each locality. Next, we extracted the minimum (tmin) and maximum (tmax) temperatures per locality, and also calculated annual range as tmax – tmin. With this microclimatic data, we re-ran the following models: (1) CTmin ~ tmin, (2) CTmax ~ tmax, and (3) Tbr ~ ar. As for macroclimatic data, when body mass had a significant effect on the thermal trait, we used the residuals of a model with just body mass as the predictor. Results are shown in Table S4.

**References**

Bentley PJ, Yorio T. 1979. Evaporative water loss in anuran amphibia: a comparative study. Comp Biochem Physiol A Physiol 62:1005–9.

Bovo RP, Navas CA, Tejedo M, Valença SE, Gouveia SF. 2018. Ecophysiology of amphibians: Information for best mechanistic models. Diversity 10:118.

Brattstrom BH. 1963. A preliminary review of the thermal requirements of amphibians. Ecology 44:238–55.

Brattstrom BH. 1968. Thermal acclimation in anuran amphibians as a function of latitude and altitude. Comp Biochem Physiol 24:93–111.

Catenazzi A, Lehr E, Vredenburg VT. 2014. Thermal physiology, disease, and amphibian declines on the eastern slopes of the Andes. Conserv Biol 28:509–17.

Christian KA, Nunez F, Clos L, Diaz L. 1988. Thermal relations of some tropical frogs along an altitudinal gradient. Biotropica 236–39.

Christian KA, Tracy CR, Tracy CR. 2017. Physical calculations of resistance to water loss improve predictions of species range models: comment. Ecology 98:2962–64.

Claussen DL. 1973. The thermal relations of the tailed frog, *Ascaphus truei*, and the Pacific treefrog, *Hyla regilla*. Comp Biochem Physiol A Physiol 44:137–53.

Colombo AF, Joly CA. 2010. Brazilian Atlantic Forest *lato sensu*: the most ancient Brazilian forest, and a biodiversity hotspot, is highly threatened by climate change. Braz J Biol 70:697–708.

Cowles RB, Bogert CM. 2006. Preliminary study of the thermal requirements of desert reptiles. Iguana 13:53–60.

Cree A. 1988. Water balance responses of the hylid frog *Litoria aurea*. J Exp Zool 247:119–25.

Edgar RC. 2004. MUSCLE: a multiple sequence alignment method with reduced time and space complexity. BMC Bioinformatics 5:1–19.

Galtier N, Nabholz B, Glémin S, Hurst GDD. 2009. Mitochondrial DNA as a marker of molecular diversity: a reappraisal. Mol Ecol 18:4541–50.

Hutchison VH, Dupré RK. 1992. Thermoregulation. Environ Physiol Amphib 206–49.

IPCC, 2014: Climate Change 2014: Synthesis Report. Contribution of Working Groups I, II and III to the Fifth Assessment Report of the Intergovernmental Panel on Climate Change [Core Writing Team, R.K. Pachauri and L.A. Meyer (eds.)]. IPCC, Geneva, Switzerland, 151 pp.

Kearney MR, Isaac AP, Porter WP. 2014. microclim: Global estimates of hourly microclimate based on long-term monthly climate averages. Sci Data 1:1–9.

Kearse M, Moir R, Wilson A, Stones-Havas S, Cheung M, Sturrock S, Buxton S, Cooper A, Markowitz S, Duran C. 2012. Geneious Basic: an integrated and extendable desktop software platform for the organization and analysis of sequence data. Bioinformatics 28:1647–49.

Kessing B, Croom H, Martin A, McIntosh C, Mcmillan WO, Palumbi S. 1989. The simple fool’s guide to PCR. Univ Hawaii Honol 17.

Kumar S, Stecher G, Tamura K. 2016. MEGA7: molecular evolutionary genetics analysis version 7.0 for bigger datasets. Mol Biol Evol 33:1870–74.

Lasiewski RC, Acosta AL, Bernstein MH. 1966. Evaporative water loss in birds—I. Characteristics of the open flow method of determination, and their relation to estimates of thermoregulatory ability*.* Comp Biochem Physiol 19:445–57.

Lutterschmidt WI, Hutchison VH. 1997. The critical thermal maximum: history and critique. Can J Zool 75:1561–74.

Lyra ML, Haddad CF, de Azeredo-Espin AML. 2017. Meeting the challenge of DNA barcoding Neotropical amphibians: polymerase chain reaction optimization and new COI primers. Mol Ecol Resour 17:966–80.

Maniatis T, Fritch EF, Sambrook J. 1982. Molecular cloning: A laboratory manual. Cold Spring Harbor Laboratory, NY. 545p.

McClanahan Jr L, Baldwin R. 1969. Rate of water uptake through the integument of the desert toad, *Bufo punctatus*. Comp Biochem Physiol 28:381–89.

Medina R, Wogan GO, Bi K, Termignoni-García F, Bernal MH, Jaramillo-Correa JP, Wang IJ, Vázquez-Domínguez E. 2021. Phenotypic and genomic diversification with isolation by environment along elevational gradients in a neotropical treefrog. *Mol Ecol* 30:4062–76.

Miller K, Packard GC. 1977. An altitudinal cline in critical thermal maxima of chorus frogs (*Pseudacris triseriata*). Am Nat 111:267–77.

Navas CA, Agudelo-Cantero GA, Loeschcke V. 2022. Thermal boldness: Volunteer exploration of extreme temperatures in fruit flies. *J Insect Physiol* 136:104330.

Navas CA, Antoniazzi MM, Carvalho JE, Suzuki H, Jared C. 2007. Physiological basis for diurnal activity in dispersing juvenile *Bufo granulosus* in the Caatinga, a Brazilian semi-arid environment. Comp Biochem Physiol A Mol Integr Physiol 147:647–57.

Oliveira-Filho AT, Fontes MAL. 2000. Patterns of floristic differentiation among Atlantic Forests in Southeastern Brazil and the influence of climate 1. Biotropica 32:793–810.

Pintanel P, Tejedo M, Ron SR, Llorente GA, Merino-Viteri A. 2019. Elevational and microclimatic drivers of thermal tolerance in Andean *Pristimantis* frogs. J Biogeogr 46:1664–75.

Rezende EL, Tejedo M, Santos M. 2011. Estimating the adaptive potential of critical thermal limits: methodological problems and evolutionary implications. Funct Ecol 25:111–21.

Riddell EA, Sears MW. 2017. Physical calculations of resistance to water loss improve species range models: reply. Ecology 98:2965–68.

Sanabria EA, Quiroga LB, Martino AL. 2012. Seasonal changes in the thermal tolerances of the toad *Rhinella arenarum* (Bufonidae) in the Monte Desert of Argentina. *J Therm Biol* 37:409–12.

Seibel RV. 1970. Variables affecting the critical thermal maximum of the leopard frog, *Rana pipiens* Schreber. Herpetologica 208–13.

Spotila JR, Berman EN. 1976. Determination of skin resistance and the role of the skin in controlling water loss in amphibians and reptiles. Comp Biochem Physiol A Physiol 55:407–11.

Terblanche JS, Deere JA, Clusella-Trullas S, Janion C, Chown SL. 2007. Critical thermal limits depend on methodological context. Proc R Soc B Biol Sci 274:2935–43.

Terblanche JS, Hoffmann AA, Mitchell KA, Rako L, le Roux PC, Chown SL. 2011. Ecologically relevant measures of tolerance to potentially lethal temperatures. J Exp Biol 214:3713–25.

Titon B, Gomes FR. 2015. Relation between water balance and climatic variables associated with the geographical distribution of anurans. Plos One 10:e0140761.

Wygoda ML. 1984. Low cutaneous evaporative water loss in arboreal frogs. Physiol Zool 57:329–37.

Young JE, Christian KA, Donnellan S, Tracy CR, Parry D. 2005. Comparative analysis of cutaneous evaporative water loss in frogs demonstrates correlation with ecological habits. Physiol Biochem Zool 78:847–56.
